# Open abdomen versus primary closure in the management of severe abdominal sepsis: What is the right way? Results of the last 5 years of a reference center

**DOI:** 10.1007/s00423-025-03693-w

**Published:** 2025-04-26

**Authors:** Tommaso Guagni, P. Prosperi, M. Marzano, A. Falcone, Matteo Bussotti, C. Bergamini, M. Mastronardi, A. Giordano

**Affiliations:** 1 Unit of Emergency Surgery, Careggi University Hospital, Florence, Italy; 2 Surgical Clinic Unit, Division of General Surgery, Department of Medical and Surgical Sciences, Hospital of Cattinara, University of Trieste, Trieste, Italy

**Keywords:** Peritonitis, Abdominal sepsis, Open abdomen, Primary closure

## Abstract

**Purpose:**

WSES guidelines allow open abdomen (OA) for critically ill patients due to secondary peritonitis in the case of inadequate source control, but this option results quite vague and with a low grade of evidence (Grade 2 C). Moreover, the emerging increasing in literature of complications, makes the use of OA in secondary peritonitis more debated. The aim of our study is to analyze the postoperative outcomes of patients undergoing OA versus primary closure (PC) in secondary peritonitis.

**Methods:**

We collected data from Tertiary Trauma Center from 2019 to 2024. The study included patients who underwent urgent laparotomy for severe secondary peritonitis, divided into two groups based on the strategy chosen in the index laparotomy: PC or OA. We retrospectively analyzed the data, considering as primary outcome post-operative mortality, while as secondary outcomes short terms complications and LOS.

**Results:**

283 patients fit the research for the diagnosis of peritonitis but only 176 were included as with a WSES-SSS > = 7. 128 patients (72,7%) were in the PC group, while 48 (27,3%) were managed with an OA strategy. There were no statistical differences in terms of mortality (*p* = 0.371), between the two groups. Complications were higher in the OA group (*p* = 0.001). From the logistic regression only MPI resulted an independent factor of mortality (*p* = 0.016; OR 1.080).

**Conclusion:**

The study shows that OA in severe secondary peritonitis does not improve mortality and is associated with higher short-term complications and incisional hernias. However, RCT are necessary to better investigate the role of OA in the management of abdominal sepsis.

## Introduction

The open abdomen (OA) technique is a life-saving option frequently associated with damage control surgery (DCS). While its role in trauma and in abdominal compartment syndrome is well described [1, 2] and represents a keystone, its use in others non-trauma patients is more controversial, especially in secondary peritonitis. However, the indication to perform OA in secondary peritonitis is still debated: on the one hand the possibility to perform OA in hemodynamically unstable patients is theorized in the main emergency guidelines [3], mainly to reduce the length of surgery interventions and allow fast internalization in intensive care units; on the other hand WSES guidelines [3] allows OA for critically ill patients due to secondary peritonitis in the case of inadequate source control or severe physiological derangement, but this option actually results quite vague and with a low grade of evidence (Grade 2 C). Especially this second option, combined with the emerging increasing in literature of complications [4–6], makes the use of OA in secondary peritonitis still more debated with the clear necessity of more specific studies. The great heterogeny in the indications of OA in clinical practice makes the final decision on which strategy must be used closely related to surgeon experience, patient’s general conditions, the severity and the origin of peritonitis, more than rigid guidelines indications.

The aim of our study is to analyze the postoperative outcomes of patients undergoing OA versus primary closure (PC) of the abdomen during secondary peritonitis.

## Materials and methods

We collected data from Emergency Tertiary Trauma Center from January 2019 to July 2024. The study group included all patients who underwent urgent laparotomy for secondary peritonitis, defined as peritoneal infection secondary to intraabdominal source of hollow viscus perforation, bowel ischemia or nonbacterial peritonitis. Database was retrospectively examinate and patients were selected according to International Classification of Disease (ICD), nineth version and clinical modification (ICD-9-CM), including all the diagnosis containing the words “peritonitis”, “perforation”, “sepsis”, “septic shock”.

### Inclusion criteria

We included all patients affected by secondary peritonitis managed either with PC or OA. From these patients we identified a subgroup of “severe abdominal sepsis” using the World Society of Emergency Surgery’s Sepsis Severity Score (WSES-SSS) [7] and we selected only those for which WSES-SSS resulted > = 7. Successively, we divided the population identified into two groups based on the strategy chosen in the index laparotomy: primary abdominal wall closure and open abdomen.

Primary abdominal closure (PC): during index laparotomy the linea alba abdominis was closed in the intent of a definitive closure; re-operation and a second laparotomy was considered on-demand when clinical, laboratories and radiological founding suggested the clinician to re-evaluate the abdominal cavity.

Open abdomen (OA): in the index laparotomy the edges of the abdominal wall were left open, and a VAC system was placed; we always used a negative wound pressure device with a suction of -20/-25 mmHg, intermittent. Patients were then transferred to intensive care units (ICU) and a second (planned) laparotomy was performed typically after 24/48 hours, according to pathological founds in the first operation, hemodynamical status, clinical evaluation on the discretion of the surgical team. According to the findings in the relaparotomy the surgeon decided to close the abdomen or repeat OA and plan a further laparotomy. All the relaparotomies after the fascia’s closure were considered on-demand and unplanned.

### Exclusion criteria

We excluded patients who underwent urgent laparotomy for other indications than suspected peritonitis (I.E. mesenteric ischemia associated with peritonitis), patients affected by pancreatitis, acute compartmental syndrome or intraabdominal hypertension, DCS conditions, trauma patients, pregnant women and age younger than 18 years.

### Data collection

Data were retrospectively collected and divided into pre-operative: demographic characteristics and indication for laparotomy; intra-operative: severity of the peritonitis using both WSES-SSS and Mannheim Peritonitis Index (MPI) [8], necessity of vasopressor, type of exudate, duration of surgery, type of closure (both for PC and definitive closure in the OA group), number of subsequent operations after index laparotomy for the OA group and, when specifically written in the notes, the reasons for choosing OA; post-operative: lactate (within the first 24 h from the operation), presence of stoma, length of stay (LOS), intra-hospital mortality, 30-days mortality, re admission within 30 days from the discharge, complications (using the Clavien-Dindo classification) [9], and the presence of incisional hernias in the follow up. Incisional hernias were diagnosed either with clinical examination or with radiological investigations. All the patients who matched the research were then revised by an external reviewer (A.G.) to closely select clinical scenario in which secondary peritonitis was the only indication for urgent laparotomy. All the surgical operations were performed by the same team, afferent to the Emergency Department and executed by either specialists or senior residents, if considered proper.

### Study outcomes

Primary outcome is post-operative mortality, while secondary outcomes include short terms complications (Clavien-Dindo 1–4) and LOS.

### Statistical analysis

Descriptive statistics and univariate analysis were conducted to describe patients’ demographics and intra-operative variables. The categorical variables were reported as percentages and absolute numbers, whereas the continuous variables were reported using medians and ranges due to their non-normal distribution (Shapiro-Wilk test). We used Mann–Whitney *U*-test to analyze continuous variables and χ^2^ or Fisher’s exact test for categorical variables. A *P* value < 0.05 was considered statistically significant (two-sided).

Univariate analyses were performed to evaluate the associations between 30-day mortality and two potential predictors: OA and the MPI. In these analyses, sex was included as a fixed factor, and age was treated as a covariate to account for potential demographic influences on mortality. The MPI was chosen as a predictor because it is a validated and widely used scoring system for assessing the severity of peritonitis and predicting outcomes, including mortality.

In this study, OA was the primary variable of interest to evaluate its association with 30-day mortality. Given the multifactorial nature of mortality, a multivariable logistic regression model was constructed to adjust for potential confounding factors, including age, sex, and the MPI, which reflect demographic and clinical characteristics. This approach allowed us to isolate the independent effect of OA on mortality while accounting for the combined influence of these variables. The statistical model subsequently underwent to the Hosmer-Lemeshow test which confirmed that the model fitted the data well. All statistical analysis were performed using the Statistical Package for Social Science (SPSS) (SPSS Inc, Chicago, Illinois, USA).

## Results

### Pre-operative

**Fig. 1:**
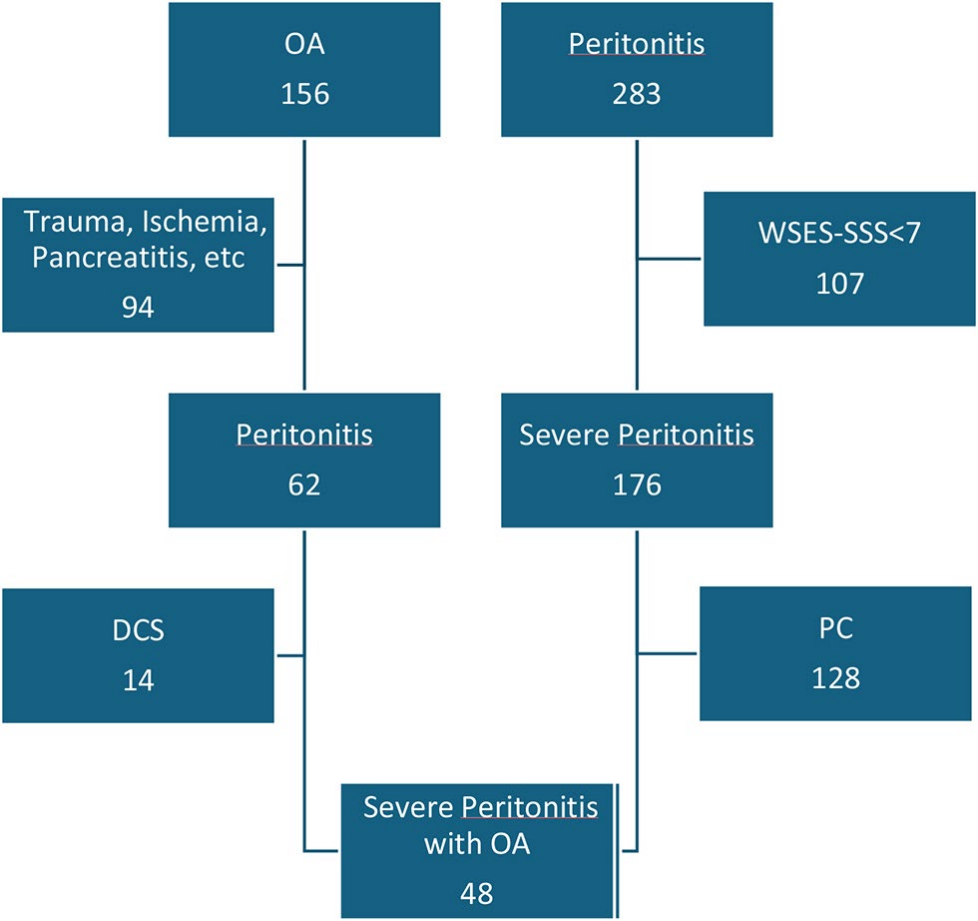
Number of OA and peritonitis selected in the study

**Fig. 2:**
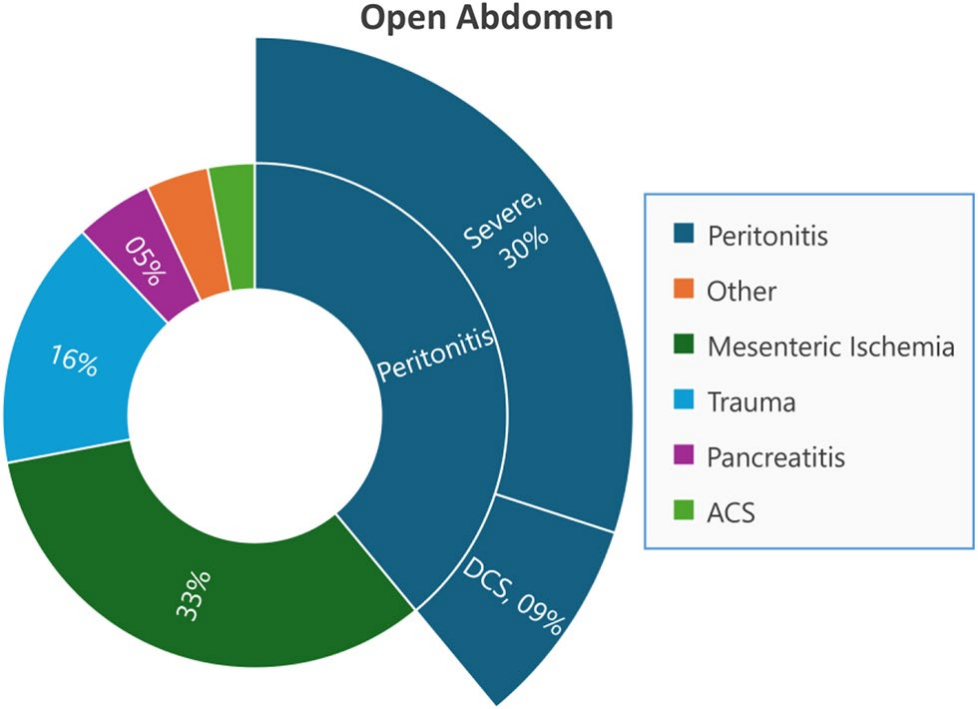
Indications for OA performed in the study period

The total number of open abdomens performed and peritonitis during this period were summarized in Figs. 1 and 2.

283 patients fitted the research for the diagnosis of “peritonitis”, “perforation”, “sepsis”, “septic shock, but only 176 were included as with a WSES-SSS > = 7. 128 patients (72,7%) were in the PC group, while 48 (27,3%) were managed with an OA strategy. Demographic characteristics and indications for laparotomy in each group are shown in Table 1.

**Table 1:** Demographic characteristics and indications for index laparotomy

	Open Abdomen	Primary Closure	*p*
	*n* = 48	*n* = 128	
Age (y)	70 (24–87)	74 (18–91)	**0.025**
Male	31 (64.6%)	70 (55.1%)	0.305
Indication for index laparotomy			0.271
Perforation	38 (79.2%)	113 (89.0%)	
Sepsis	5 (10.4%)	7 (5.5%)	
Ischemia	3 (6.3%)	3 (2.4%)	
Occlusion	2 (4.2%)	4 (3.1%)	

### Intra-operative

As previously mentioned, we considered the WSES-SSS as a filter for severe peritonitis, the median of the score was 10 for both groups; MPI was higher in the OA group (32 Vs 30, *p* = 0.030), but patients in the OA management were more likely to necessitate of vasopressors at the very end of the intervention (89.6 Vs 48.8%, *p* < 0.001). Enteric exudate reclaimed more often OA than PC (60.4 Vs 47.2%, *p* = 0.130), but this data does not reach statistical relevance. However, duration of surgery was significantly longer in the PC group: 70 Vs 120 min, *p* < 0.001. On the contrary, we found that fascia closure rate resulted higher in the PC group (96.9 Vs 68.8% *p* < 0.001) and that 25% of the patients who underwent OA strategy received only skin closure, while meshes were used three times but only in the OA group.

In the OA group we reported a median of 1 planed re-laparotomy, but we underline a range that went from 0 (patients who died before the second look) to 6. The indications for choosing an OA were 35.4% for abdominal contamination, 22.9% for hemodynamical instability at the end of the procedure, 10.4% for the intention to delay anastomosis, while in 31.3% of the cases the reason for OA was not specified in the operative notes. Intra-operative variables are listed in Table 2.

**Table 2:** Intra-operative variables

	Open Abdomen	Primary Closure	*p*
	*n* = 48	*n* = 128	
WSES-SSS	10 (7–15)	10 (7–15)	0.088
MPI	32 (17–39)	30 (21–43)	**0.030**
Vasopressor	43 (89.6%)	62 (48.8%)	**< 0.0001**
Type of exudate			0.130
Purulent	19 (39.6%)	67 (52.8%)	
Enteric	29 (60.4%)	60 (47.2%)	
Indication for OA			
Hemodynamical instability	11 (22.9%)		
Contamination	17 (35.4%)		
Delay anastomosis	5 (10.4%)		
Not specified	15 (31.3%)		
N. OA revisions	1 (0–6)		
Modality of closure			**< 0.0001**
Fascia closure	33 (68.8%)	123 (96.9%)	
Only skin closure	12 (25%)	4 (3.1%)	
Mesh	3 (6.3%)	0	
Operative time (min)	70 (45–190)	120 (45–250)	**< 0.0001**

### Post-operative

There were no statistical differences in terms of mortality between the two groups; in particular: 30-days mortality resulted 39.6% in the OA and 31.5% in the PC group (*p* = 0.371), in-hospital mortality was 41.7% and 32.3% respectively (*p* = 0.287). Early readmission (within 30 days from the discharge) was 4.2% in the OA and 3.2% in the PC (*p* = 0.668). There were no differences in lactates (17 mg/dL in OA Vs 18 mg/dL in PC, *p* = 0.870) but the range resulted extremely wild for both cohorts: 2-180 mg/dL for OA group and 1-477 for PC group. Stoma rate resulted substantially the same in OA and in PC (63.8 Vs 63%, *p* = 1.000) and investigating those patients who were managed with OA expressively for demanding anastomosis, we found that all the patients but one underwent stoma creation. LOS as well was not significantly different between the two cohorts: 18 for the OA, 14 for the PC, *p* = 0.128.

Unplanned re-laparotomy resulted more frequent in the OA cohort (22.9%) than in the PC one (8.7%) with *p* = 0.019. Incisional hernias were detached in the 9.6% of the patient who underwent PC and in the 57.9% (*p* < 0.0001) of the patients in the OA section, the data considered also those patients in the OA group managed with only skin closure for the impossibility to synthetize abdominal fascia due to margin retraction.

We used the Clavien-Dindo classification for complicacies, and we reported a statically relevant difference (*p* = 0.001) between the two groups, with a consistent result against OA both for CD II and for CD III-IV-V; CD I were instead higher in the PC group. Post-operative characteristics are shown in Table 3.

**Table 3:** Post-operative variables

	Open Abdomen	Primary Closure	*p*
	*n* = 48	*n* = 128	
Lactate (mg/dL)	17 (2-180)	18 (1-477)	0.870
Unplanned re-laparotomy	11 (22.9%)	11 (8.7%)	**0.019**
Stoma rate	30 (63.8%)	80 (63.0%)	1.000
LOS	18 (2–94)	14 (1–70)	0.128
Clavien-Dindo			**0.001**
1	1 (2.1%)	23 (18.1%)	
2	11 (22.9%)	14 (11.0%)	
3a	5 (10.4%)	9 (7.1%)	
3b	5 (10.4%)	2 (1.6%)	
4	2 (4.2%)	6 (4.7%)	
5	17 (35.4%)	36 (28.3%)	
Medical complications			
Cardiovascular	17 (35.4%)	31 (24.2%)	
Respiratory	18 (37.3%)	48 (38.0%)	
Renal	19 (39.6%)	12 (16.4%)	
Neurologic	8 (16.7%)	4 (16.4%)	
In-hospital mortality	20 (41.7%)	41 (32.3%)	0.287
30-days mortality	19 (39.6%)	40 (31.5%)	0.371
30-days readmission	2 (4.2%)	4 (3.2%)	0.668
Incisional hernia	22 (57.9%)	7 (9.6%)	**< 0.0001**

The univariate analysis conducted to assess the influence of OA on 30-day mortality, using sex as fixed factor, and age as covariate, showed that those factors were not statistically significant contributors to the variability in the model (OA *p* = 0.213; sex *p* = 0.242; age *p* = 0.064). Similarly, the univariate analysis conducted with MPI showed that MPI, sex and age were not statistically significant contributors to the variability in the model (MPI *p* = 0.917; sex *p* = 0.394; age *p* = 0.104).

Although the univariate analysis showed that neither OA, MPI, age, nor sex were statistically significant predictors of 30-day mortality, a multivariable logistic regression was performed based on the clinical relevance of these variables. Univariate p-values were not used to determine inclusion in the multivariable model, as variables that may not appear significant in univariate analysis can still have important predictive or confounding effects when combined with other factors.By including OA, MPI, age, and sex, we aimed to account for potential confounding, explore their combined influence, and assess their interactions to better understand their collective impact on 30-day mortality. This approach ensures that clinically plausible variables were adequately considered, maximizing the robustness of the analysis.

**Fig. 3:**
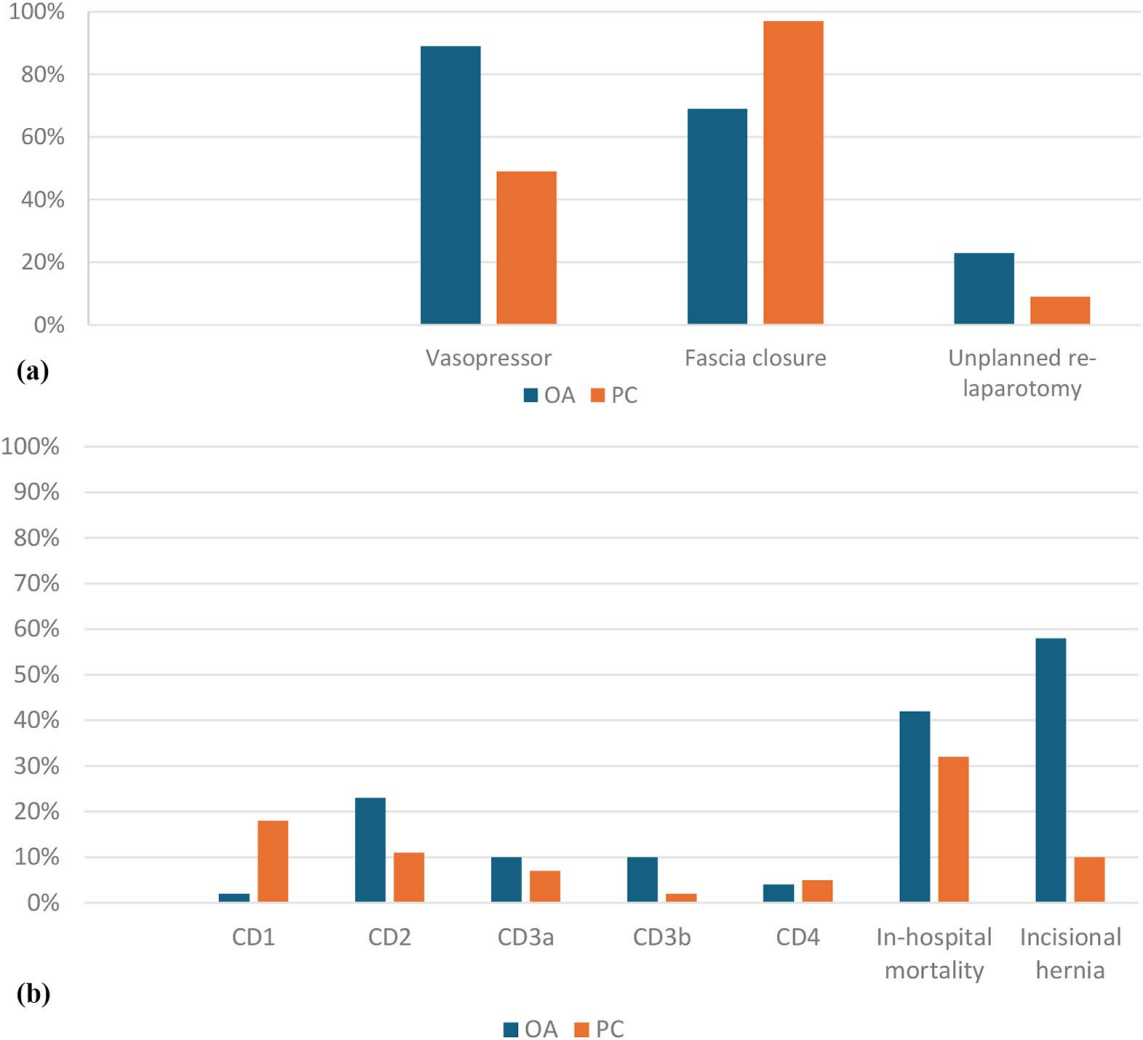
**a**) Statistically significant results. OA = Open Abdomen; PC = Primary closure. **b**) Statistically significant results; OA = Open Abdomen; PC = Primary closure

**Fig. 4:**
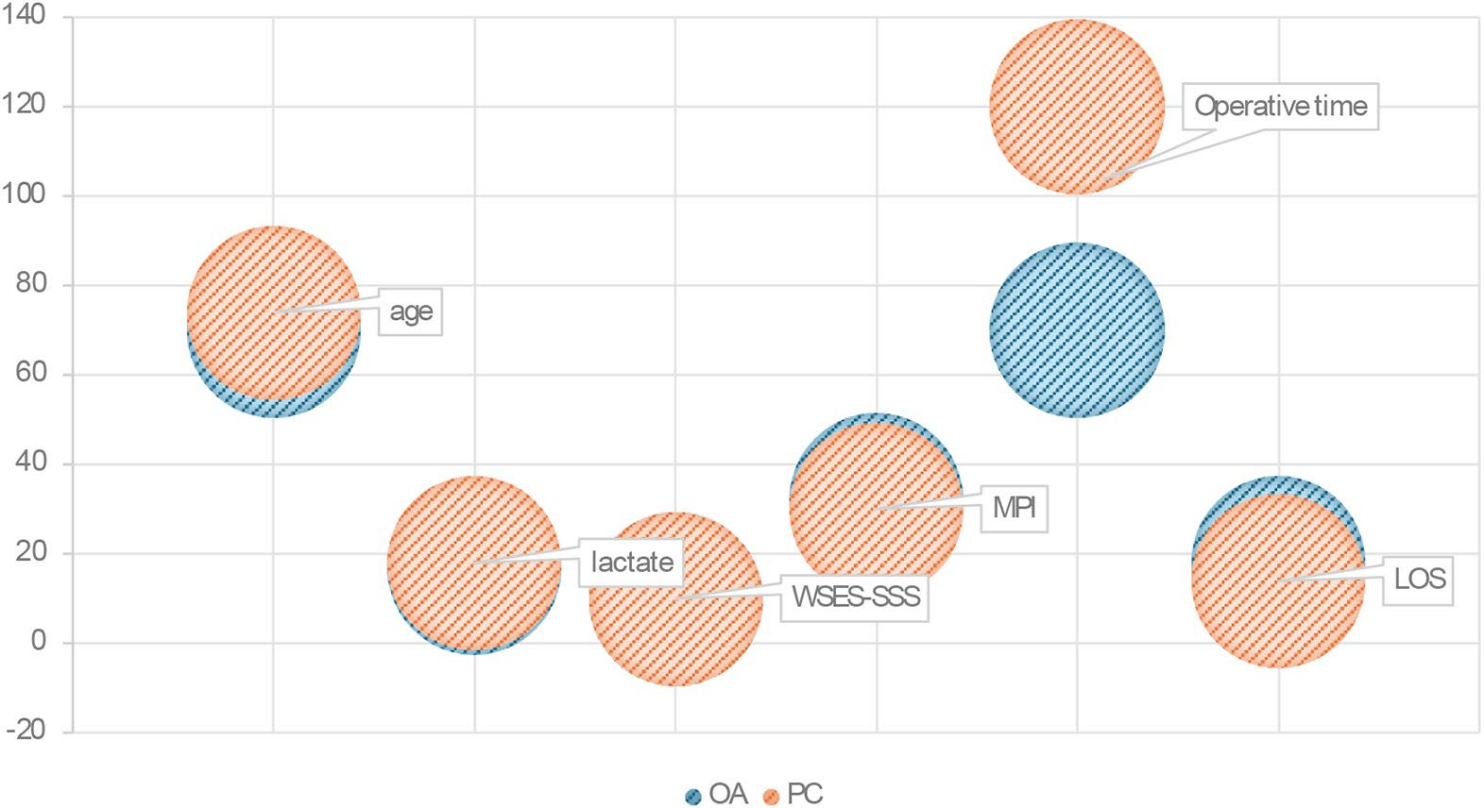
Distribution of pre, intra and post-operative variables

From the multivariable logistic regression model only MPI resulted an independent factor of mortality (*p* = 0.016; OR 1.080); complete logistic regression is shown in Table 4. All statistically significant results all reported in Fig. 3a and b, while Fig. 4 shows the distribution of pre, intra and post-operative variables.

**Table 4:** Logistic regression. OR = Odds ratio

	*p*	OR	95% C.I.for OR	
			Inferior	Superior
OA	0,336	1,448	0,681	3,077
MPI	0,016	1,080	1,014	1,150
Age	0,123	1,021	0,994	1,048
Sex	0,651	1,177	0,581	2,383

## Discussion

The management of abdominal sepsis is complex and multimodal and the standard of care for these patients is in continuous evolution, moreover, this condition is often associated with multiple variables like physiologic derangements or inadequate source control which makes the management even more challenging. In this scenario, we are aware that the comparison between OA and PC represents just one of the chapters. The use of OA in non-trauma patients is theorized in WSES guidelines, as previously mentioned [3], under some circumstances: physiological derangement, the need for a deferred intestinal anastomosis, visceral oedema with the risk of ACS and a failure to source control which involves a persistent source of infection. However, these are low grade of recommendation (Grade 2 C) and there are no definitive data regarding an improvement in survival connected to the use of OA in this scenario. From the listed indications to perform OA in peritonitis, we dwelled on “failure to source control” which in our opinion resulted the more specific in an abdominal sepsis scenario. Although the effort to clearly define the “adequate source control” in which OA may be used [10], in clinical practice the lack of definitive guidelines could expose to a misuse of this strategy.

In our monocentric, retrospective analysis we found no differences in terms of mortality (both in-hospital and at 30 days) between the two groups (see Fig. 3b). Furthermore, we reported a high mortality rate in both OA (41.7%, in hospital) and PC (32.3%, in hospital), this result may be addressed to the fact that we enrolled only severe peritonitis, which substantially included abdominal sepsis in immunodeficient and/or old patients. The logistic regression as well showed that the use of OA was not associated with a reduction of mortality (*p* = 0.336; OR 1,448), thus was not a protective factor against mortality.

Our results resulted consistent with the literature, although the scarcity of recent studies makes comparison difficult. In 2019, Kao [11] demonstrated a disadvantage in terms of mortality with the use of OA compared to PC. In their recent analysis, the authors compared the two cohorts of OA and PC using propensity score matching, concluding that the use of OA in secondary peritonitis not only is clearly unfavorable, but it is also affected by a higher incidence of complications. However, this study included a wide variety of clinical situations and severities of peritonitis. Additionally, although it was rightly addressed in the discussion, different surgical specialties certainly have different approaches in the management of secondary peritonitis, which could lead to bias in the indication for OA. In contrast, our analysis emphasizes that is the same highly trained group of surgeons in emergency surgery which is responsible for the indication.

The same conclusions were reported in a large study by Nzenwa [12], in which propensity matching was used as well, concluding that mortality was statistically lower in the PC group. However, it is to notice that in this study there was a significant difference between the ‘unmatched’ population and the population selected after propensity scoring, suggesting extremely high variability within the population itself and introducing the possibility of residual bias.

We decided to use the WSES-SSS to determinate the severity of the sepsis as it was the most recent and validated scoring system specifically designed for abdominal sepsis [7, 13]. WSES-SSS authors found a statistical relevant increase in mortality with the increase of the score: from 6.3% forthose who had a score of 4–6, to 41.7% forthose who had a score of ≥ 7. Our analysis revealed that OA was used more frequently in peritonitis with a higher MPI and WSES-SSS values, suggesting a larger use in more severe clinical scenarios. Even if this finding was not showed in the univariate analysis, it was also demonstrated in the multivariable logistic regression model, where higher MPI values appear to correlate more with 30-day mortality (p = 0.016; OR 1.080). Furthermore, the procedures in which OA was chosen as a surgical strategy were statistically shorter in surgical time (p < 0.0001) as shown in Fig. 4. These data indicated that, in our experience, the choice to use OA was driven by the severity of the peritonitis. Although cases of OA as ‘pure’ damage control surgery were not included in the study, as this use is well-defined in international guidelines [2, 3] for non-traumatic patients, it emerges that our ‘guideline’ was to assimilate the most serious patients within the cohort of severe peritonitis to those deserving DCS, essentially creating a usage that we could define as “near DCS”. Strictly connected with this finding, recent literature highlighted the role of direct peritoneal resuscitation (DPR) as an adjunctive treatment in the management of severe sepsis [14]. In fact, DPR shown promising results in the latter studies [15] for enhancing hemodynamic stability and improving outcomes in severe sepsis and peritonitis; deserving a promising major role in the complex and multimodal management of severe abdominal sepsis.

The most frequent indication for OA was for abdominal contamination with the aim of improving source control; however, as noted, there was no demonstrable improvement in outcomes regarding mortality, LOS (*p* = 0.128), and readmissions (*p* = 0.668). In contrast, there was a lower incidence of unplanned relaparotomies in the PC group compared to the OA group, see Fig. 3. Van Ruler [16] in 2007 published one of the few RCTs on this topic, comparing on-demand laparotomies versus scheduled ones in severe peritonitis; this study resulted certainly one of the cornerstones in literature regarding the topic and concludes, in line with our results, that there were no significant differences between the two groups in terms of mortality, and that unplanned relaparotomies were also lower in the PC group. Although this study was statistically significant, it was not recent, and other works, such as that by Bleszynski [17], reported a benefit in using OA both in terms of survival and in the number of unplanned relaparotomies. In particular, the failure of PC that subsequently led to reoperation was due to the progression of sepsis or, in some cases, to secondary complications such as compartment syndrome.

In 10.4% of cases, OA was adopted with the specific indication to delay anastomosis, aiming to avoid stoma creation. Interestingly, of all the patients (5) treated with this intent, only one underwent anastomosis and avoided stoma; moreover, stoma rate was essentially identical in the two groups. This finding was certainly related to specific sites of sepsis origin (i.e. diverticular perforations) or clinical scenarios (i.e., anastomotic dehiscence), where, although conceptually gaining time to reduce inflammation and edema of the small gut or colon could favor an OA strategy, in clinical practice, it still leads to stoma creation. This data resulted difficult to compare in the literature, as specific studies on the use of OA in secondary peritonitis were not numerous, and stoma rates were not always reported. In a recent (2023) work by Peng [18] stoma rate was found to be comparable between the OA and PC groups (35%), but the patients were not specifically selected for sepsis; rather, they were more generally classified as non-trauma, mainly including intestinal ischemia or compartment syndromes. Nonetheless, this resulted aligned with what emerged from our analysis, although stoma rate resulted lower.

In terms of complications, there was a statistically significant difference (*p* = 0.001) between the two groups in favor to PC. We reported one case of low-output entero-atmospheric fistula in a particularly complex, multiply operated patient, who was managed with nutritional support and subsequent reoperation, requiring a long hospital stay (94 days). We did not report any cases of compartment syndrome, although intra-abdominal pressure measurements were not systematically performed on all patients and, consequently, are not included in the results of our analysis.

The difference in the incidence of incisional hernias was highly statistically significant between the two groups (*p* < 0.0001) in favor to the PC group (9.6% vs. 57.9%). This stark difference was partly due to a notable number of patients in the OA group who had only skin closure (25%), thus planning for a ventral hernia, but it still reflected a problem to be considered when deciding to postpone the primary fascia closure at the end of the index laparotomy.

Literature reported variable data on the incidence of incisional hernias, which were nonetheless consistent with our findings and, in any case, unfavorable to OA. Theodorou in 2021 [19] reported an incidence of incisional hernias between 5% and 20% in patients undergoing PC, compared to a rate between 35% and 65% in those undergoing OA, regardless the indications. As highlighted by several reviews [20–22] different methods of fascia closure in the aim to prevent incisional hernias could lead to varying outcomes. For example, three studies [23–25] two of which were prospective, reported long-term outcomes, with the incidence of incisional hernias post-OA varying from 21% at 21 months to 54% at 5 years, using a sutured bridging prosthesis on the fascial margins during OA, associated with NPWT.

Literature also reported varying incidences of incisional hernias based on the type of TAC device used, highlighting expectations for newer dynamic traction closure systems (Wittmann patch or ABRA system) [26, 27] In our experience, we always used a standard negative pressure system and rarely (only three cases in the OA group and none in the PC group) we utilized meshes, but no dynamic traction closure systems. Specifically, in the case of OA in contaminated fields such as abdominal sepsis, the choice to apply prostheses for prophylactic closure or in cases of inability to achieve primary closure due to extreme retraction of margins, becomes even more complex, limiting the choice to biological or biosimilar prostheses, which, however, still have limited use in literature, also due to high costs [20, 28].

We underline that our study had several limitations: first, it was a monocentric, retrospective study based on the experience of a single surgical team. Additionally, while the sample size was not small, it was certainly different between the two cohorts, and there was a significant difference in median ages. Similarly, there was heterogeneity in both groups regarding the types of interventions performed, ranging from peptic ulcer sutures to colonic resections. However, the site of perforation was considered in determining the severity of peritonitis in the main scores used (WSES-SSS and MPI), and, in line with the literature [7, 13, 29–31], we considered that the site of origin of the sepsis resulted more determinative for the considered outcomes than the type of intervention performed. Moreover, from a statistical standpoint, except for age, the two groups appeared homogeneous in their preoperative characteristics.

Unfortunately, due to the wideness and heterogeneousness of the population considered, we did not have accurate data regarding time between symptoms presentation and surgery and this lack of information represented a deficiency, thus it must be addressed as a major bias as delay in surgical management could affect mortality.

Another limitation was the variable follow-up period, especially when concerning incisional hernias as a long-term complication. Considering that we enrolled patients from 2019 to the present, it is true that the observation period could be considered adequate for the outcomes analyzed; however, targeted studies on long-term complications are necessary.

Finally, although we assumed that having a single group dedicated to emergency surgery was a homogenizing factor in the choice between OA and PC, there was still a certain degree of individual variability, related on surgeon’s experience, in the choice of surgical strategy that must be regarded as a bias. Evidence of this bias was, for example, the fact that in one-third of patients with OA, the reason for this choice was not specified, making it effectively subjective.

It is known that designing prospective or randomized studies in the field of emergency surgery is challenging, although such works are necessary to better define the role of OA in secondary peritonitis as well. High hopes are placed on the COOL trial [32] a RCT that has been ongoing since 2018, which has been collecting data from around the world and from which systematic answers are expected regarding the best management of abdominal sepsis.

## Conclusions

The study shows that OA in severe secondary peritonitis does not improve mortality and is associated with higher short-term complications and incisional hernias rates. We deem that the use of OA remains a strategy to consider in patients with severe clinical conditions and abdominal sepsis, particularly as a “near DCS” approach where time is a critical factor. In this regard, it remains undeniable that the use of an OA strategy can provide surgeons with more time to better weigh the definitive treatment of secondary peritonitis, although predictive variable of postoperative mortality may be linked to the severity of the peritonitis itself.

It is known that designing prospective or randomized studies in the field of emergency surgery is challenging, although such works are necessary to better define the role of OA in secondary peritonitis as well. High hopes are placed on the COOL trial a RCT that has been ongoing since 2018, which is collecting data from around the world and from which systematic answers are expected regarding the best management of abdominal sepsis.

## Data Availability

No datasets were generated or analysed during the current study.

## References

[1] Roberts DJ, Leppäniemi A, Tolonen M, Mentula P, Björck M, Kirkpatrick AW et al (2023) The open abdomen in trauma, acute care, and vascular and endovascular surgery: comprehensive, expert, narrative review. BJS Open.;7 10.1093/bjsopen/zrad084 PMC10601091 37882630

[2] Chiara O, Cimbanassi S, Biffl W, Leppaniemi A, Henry S, Scalea TM et al (2016) International consensus conference on open abdomen in trauma. Journal of Trauma and Acute Care Surgery.;80:173–83 10.1097/TA.0000000000000882 27551925

[3] Coccolini F, Roberts D, Ansaloni L, Ivatury R, Gamberini E, Kluger Y et al (2018) The open abdomen in trauma and non-trauma patients: WSES guidelines. World J Emerg Surg 13:7 29434652 10.1186/s13017-018-0167-4 PMC5797335

[4] Prete F, De Luca GM, Pasculli A, Di Meo G, Poli E, Sgaramella LI et al (2022) Retrospective study of indications and outcomes of open abdomen with negative pressure wound therapy technique for abdominal sepsis in a tertiary referral centre. Antibiotics 11:1498 36358153 10.3390/antibiotics11111498 PMC9686976

[5] Coccolini F, Montori G, Ceresoli M, Catena F, Ivatury R, Sugrue M et al (2017) IROA: international register of open abdomen, preliminary results. World J Emerg Surg 12:10 28239409 10.1186/s13017-017-0123-8 PMC5320725

[6] Coccolini F, Montori G, Ceresoli M, Catena F, Moore EE, Ivatury R et al (2017) The role of open abdomen in non-trauma patient: WSES consensus paper. World J Emerg Surg 12:39 28814969 10.1186/s13017-017-0146-1 PMC5557069

[7] Abdel-Kader S, Sartelli M, Abu-Zidan F (2019) Complicated intra-abdominal infections: a prospective validation study of the WSES sepsis severity score. Singap Med J 60:317–321 10.11622/smedj.2018120 PMC6595066 30311628

[8] Neri A, Fusario D, Marano L, Savelli V, Bartalini Cinughi de Pazzi A, Cassetti D et al (2020) Clinical evaluation of the Mannheim prognostic index in post-operative peritonitis: a prospective cohort study. Updates Surg 72:1159–1166 32578039 10.1007/s13304-020-00831-5

[9] Clavien PA, Sanabria JR, Strasberg SM (1992) Proposed classification of complications of surgery with examples of utility in cholecystectomy. Surgery 111:518–526 1598671

[10] Coccolini F, Sartelli M, Sawyer R, Rasa K, Viaggi B, Abu-Zidan F et al (2023) Source control in emergency general surgery: WSES, GAIS, SIS-E, SIS-A guidelines. World J Emerg Surg 18:41 37480129 10.1186/s13017-023-00509-4 PMC10362628

[11] Kao AM, Cetrulo LN, Baimas-George MR, Prasad T, Heniford BT, Davis BR et al (2019) Outcomes of open abdomen versus primary closure following emergent laparotomy for suspected secondary peritonitis: A propensity-matched analysis. J Trauma Acute Care Surg 87:623–629 31045736 10.1097/TA.0000000000002345

[12] Nzenwa IC, Rafaqat W, Abiad M, Lagazzi E, Panossian VS, Hoekman AH et al (2024) The open abdomen after Intra-Abdominal contamination in emergency general surgery. J Surg Res 301:37–44 38909476 10.1016/j.jss.2024.05.037

[13] Sartelli M, Abu-Zidan FM, Catena F, Griffiths EA, Di Saverio S, Coimbra R et al (2015) Global validation of the WSES sepsis severity score for patients with complicated intra-abdominal infections: a prospective multicentre study (WISS Study). World J Emerg Surg 10:61 26677396 10.1186/s13017-015-0055-0 PMC4681030

[14] Smith JW, Neal Garrison R, Matheson PJ, Harbrecht BG, Benns MV, Franklin GA et al (2014) Adjunctive treatment of abdominal catastrophes and sepsis with direct peritoneal resuscitation. J Trauma Acute Care Surg 77:393–399 25159241 10.1097/TA.0000000000000393

[15] Okumura K, Latifi R, Smiley A, Lee JS, Shnaydman I, Zangbar B et al (2022) Direct peritoneal resuscitation (DPR) improves acute physiology and chronic health evaluation (APACHE) IV and acute physiology score when used in damage control laparotomies: prospective cohort study on 37 patients. Surg Technol Online.;41 10.52198/22.STI.41.GS1620 36041078

[16] van Ruler O, Mahler CW, Boer KR, Reuland EA, Gooszen HG, Opmeer BC et al (2007) Comparison of On-Demand vs planned relaparotomy strategy in patients with severe peritonitis. JAMA 298:865 17712070 10.1001/jama.298.8.865

[17] Bleszynski MS, Chan T, Buczkowski AK (2016) Open abdomen with negative pressure device vs primary abdominal closure for the management of surgical abdominal sepsis: a retrospective review. Am J Surg 211:926–932 27020900 10.1016/j.amjsurg.2016.01.012

[18] Peng CC, Tay J, Tham N, Tully EK, Shakerian R, Furlong T et al (2023) Use of temporary abdominal closure in Non-Trauma surgery: A cohort study. World J Surg 47:1477–1485 36847850 10.1007/s00268-023-06960-3

[19] Theodorou A, Jedig A, Manekeller S, Willms A, Pantelis D, Matthaei H et al (2021) Long term outcome after open abdomen treatment: function and quality of life. Front Surg.;8 10.3389/fsurg.2021.590245 PMC8039509 33855043

[20] Deng Y, Ren J, Chen G, Li G, Guo K, Hu Q et al (2016) Evaluation of polypropylene mesh coated with biological hydrogels for temporary closure of open abdomen. J Biomater Appl 31:302–314 27114442 10.1177/0885328216645950

[21] van Boele P, Wind J, Dijkgraaf MGW, Busch ORC, Carel Goslings J (2009) Temporary closure of the open abdomen: A systematic review on delayed primary fascial closure in patients with an open abdomen. World J Surg 33:199–207 19089494 10.1007/s00268-008-9867-3 PMC3259401

[22] Miller PR, Thompson JT, Faler BJ, Meredith JW, Chang MC (2002) Late fascial closure in lieu of ventral hernia: the next step in open abdomen management. J Trauma: Injury Infect Crit Care 53:843–849 10.1097/00005373-200211000-00007 12435933

[23] Willms A, Güsgen C, Schaaf S, Bieler D, von Websky M, Schwab R (2015) Management of the open abdomen using vacuum-assisted wound closure and mesh-mediated fascial traction. Langenbecks Arch Surg 400:91–99 25128414 10.1007/s00423-014-1240-4

[24] Petersson U, Acosta S, Björck M (2007) Vacuum-assisted wound closure and Mesh‐mediated fascial Traction—A novel technique for late closure of the open abdomen. World J Surg 31:2133–2137 17879112 10.1007/s00268-007-9222-0

[25] Bjørsum-Meyer T, Skarbye M, Jensen KH (2013) Vacuum with mesh is a feasible temporary closure device after fascial dehiscence. Dan Med J 60:A4719 24192239

[26] Abosena W, Tedesco A, Han SM, Bugaev N, Hojman HM, Johnson BP et al (2024) A Cost-Effectiveness analysis of Wittmann Patch-Assisted abdominal closure compared to planned ventral hernia in management of the open abdomen. Am Surg 90:1140–1147 38195166 10.1177/00031348241227214

[27] Nemec HM, Benjamin Christie D, Montgomery A, Vaughn DM (2020) Wittmann patch. Am Surg 86:981–984 32779473 10.1177/0003134820942156

[28] Köckerling F, Alam NN, Antoniou SA, Daniels IR, Famiglietti F, Fortelny RH et al (2018) What is the evidence for the use of biologic or biosynthetic meshes in abdominal wall reconstruction? Hernia 22:249–269 29388080 10.1007/s10029-018-1735-y PMC5978919

[29] Tolonen M, Sallinen V, Leppäniemi A, Bäcklund M, Mentula P (2019) The role of the intra-abdominal view in complicated intra-abdominal infections. World J Emerg Surg 14:15 30976292 10.1186/s13017-019-0232-7 PMC6441193

[30] Sartelli M, Coccolini F, Kluger Y, Agastra E, Abu-Zidan FM, Abbas AES et al (2021) WSES/GAIS/SIS-E/WSIS/AAST global clinical pathways for patients with intra-abdominal infections. World J Emerg Surg 16:49 34563232 10.1186/s13017-021-00387-8 PMC8467193

[31] Sartelli M, Chichom-Mefire A, Labricciosa FM, Hardcastle T, Abu-Zidan FM, Adesunkanmi AK et al (2017) The management of intra-abdominal infections from a global perspective: 2017 WSES guidelines for management of intra-abdominal infections. World J Emerg Surg 12:29 28702076 10.1186/s13017-017-0141-6 PMC5504840

[32] Kirkpatrick AW, Coccolini F, Tolonen M, Minor S, Catena F, Gois E et al (2023) The unrestricted global effort to complete the COOL trial. World J Emerg Surg 18:33 37170123 10.1186/s13017-023-00500-z PMC10173926

